# Prevalence and associated factors of visual impairment among adults at Debre Berhan town, North Shewa, Ethiopia

**DOI:** 10.1186/s12886-020-01586-8

**Published:** 2020-08-03

**Authors:** Natnael Lakachew Assefa, Addisu Wondifraw Admas, Nebiyat Feleke Adimasu

**Affiliations:** 1 Department of Optometry, School of Medicine, College of Medicine and Health Science, University of Gondar, Gondar, Ethiopia; 2 Debre Berhan Referral Hospital, North Shewa, Ethiopia

**Keywords:** Visual impairment, Associated factors, Adults, Debre Berhan, Ethiopia

## Abstract

**Background:**

Visual impairment refers to presenting distance visual acuity worse than 6/18 in the worst eye. It remains a global challenge that greatly affects the mobility, social participation and the quality of life of the people. This study was aimed to determine the prevalence and associated factors of visual impairment among adults aged ≥ 18 years.

**Methods:**

A community-based cross-sectional study was conducted at Debre Berhan town. Systematic random sampling method was employed to select the study participants from adult’s aged ≥18 years. Data was collected by interview with a pre-tested semi structured questionnaire. Both anterior and posterior segment ocular examinations were done by Optometrists and Ophthalmologist. After all ocular examinations adult’s aged ≥18 years with presenting Visual acuity of < 6/18 in the worst eye were considered as visually impaired. Multivariate logistic regression was used to identify the determinant factors and *p* value less than 0.05 was considered as statistically significant.

**Results:**

A total of 416 participants were enrolled in the study with a 98.6% response rate. The prevalence of visual impairment among adults aged ≥ 18 years was 16.8% (95% CI, 13.5–20.2%). Among the overall prevalence of visually impaired adults 27 (6.49%) had bilateral VI and 43 (10.34%) had monocular VI. Aged > 64 years (AOR = 12.18, 95%CI: 4.47–33.20), illiterates AOR = 3.02, 95% CI: 1.36–6.72), previous eye trauma (AOR = 4.44, 95% CI: 1.64–12.04), family size > 5 (AOR: 4.44, 95% CI: 1.43–13.75) and family history of eye problem (AOR = 7.02, 95% CI: 1.95–25.22) had statistically significant association with visual impairment.

**Conclusions:**

Prevalence of visual impairment among adults was found to be a significant public health problem. Older age, illiterates, previous eye trauma, large family size and family history eye problem were positively associated with visual impairment.

## Background

Visual impairment (VI) refers to a functional limitation of the eye or visual system due to a disorder or disease that results in poor vision in the worst eye. According to World Health Organization (WHO) revised definition, it is defined as presenting distance visual acuity worse than 6/18 in the worst eye [1]. Classification of severity of VI recommended by the Resolution of the International Council of Ophthalmology and WHO Consultation includes Moderate VI, Severe VI and blindness based on presenting VA worse than 6/18, 6/60, and 3/60 respectively [1, 2]. Among the global population 216·6 million were moderate or severe VI. The leading causes were uncorrected refractive error (116·3 million), cataract (52·6 million), age-related macular degeneration (8·4 million), glaucoma (4·0 million), and diabetic retinopathy (2·6 million) [3]. The prevalence of VI among adults aged 40 years and above in the South Indian State of Andhra Pradesh was 14.3% [4], In Saudi among adults aged ≥18 years was (23.5%) [5], in East Delhi district among adults aged ≥ 40 years and above was 11.4% [6] and in rural area of Coastal Karnataka state among adults aged ≥18 years was 25.7% [7]. Based on the presenting visual acuity (PVA) prevalence of VI was different in African countries. In Ghana among Cocoa Farmers aged ≥ 40 years was 22.7% [8], In Upper Egypt among adults aged ≥ 40 years was 38.8% [9] and In Southern Sudan among aged ≥ 5 years was 11.8% [10]. Based on 2006 national survey blindness and low vision were major public health problems in Ethiopia. Based on presenting vision in the better eye the national prevalence of blindness and low vision was 1.6 and 3.7% respectively. The major causes of low vision were cataract (42.3%), refractive error (33.4%), trachomatous corneal opacity (7.7%), other corneal opacity (5.9%) and macular degeneration (4.6%) [11].

VI remains to be a major public health problem especially in low and middle income countries which was estimated to be four times higher than in high-income countries [12]. VI affects the quality of life and socio-economic characteristics of the people like mobility, social participation and find a job [13]. Hence, their ability to find employment and support themselves and provide for their families is diminished [14]. However, there was no previous study on the prevalence and associated factors of VI among adults at a community level in Ethiopia as well as in the study area. There is limited access to eye care service for the large number of populations at Debre Berhan town in which only one eye care service center and few eye care professionals are providing services for more than about 80,000 populations. So the current study aimed to estimate the total magnitude of VI at the town and it might be helpful for health authorities to plan strategies for eye care services in the study area.

## Methods

### Study design, setting and sampling

A community-based cross-sectional study was conducted at Debre Berhan town from April 30, 2018 to May 15, 2018. Debre Berhan town is located in North Shewa, Amhara Regional State, 120 km away from Addis Ababa (the capital city of Ethiopia) in the north direction and 688 km from Bahir Dar (capital city of Amhara National Regional State). It has 88,375 total populations (39,961 males and 48, 414 females), of which 64.4% are adults aged ≥18 years (unpublished data obtained from Debre Berhan town woreda health office). All adults aged ≥ 18 years who lived at Debre Berhan town for at least 6 months were the source and study population.

Sample size was determined with single population proportion formula. $n=\frac{{\left({Z}_{\alpha /2}\right)}^2P\left(1-P\right)}{d^{2.}}$ (*n =* Sample size, Z = The Value of z statistic at 95% confidence level = 1.96, P – Proportion of visual impairment = 50% = 0.5 (Since community based study on the presenting visual acuity was not conducted in the study area or other similar areas which had related population characteristics and methodology of the current study, 50% proportion was used), d – Maximum tolerable error (marginal error) 5% = 0.05, *n* = 384). By adding 10% nonresponse rate, the final sample size was estimated at 422. In the study area, there were 20,770 households and nine kebeles (administrative groups). All administrative kebeles were included in the study by proportionally allocating the households in each kebele according to their size. Systematic random sampling method was employed to select the households by using an interval of constant (k = 49, K was calculated as the total households [20,770] divided by sample size [422]). If more than one eligible adult’s aged ≥18 years were found in the selected household, a lottery method was used to recruit the sample.

### Operational definitions

#### Visual impairment

VI was defined as presenting distance visual acuity worse than 6/18 to no light perception (NLP) in the worst eye. It was further classified into moderate VI (Presenting visual acuity (PVA) < 6/18 - ≤ 6/60), severe VI (PVA < 6/60 - ≤ 3/60), blindness (PVA < 3/60 - NLP), monocular moderate VI (PVA of < 6/18 - ≤ 6/60 in one eye and 6/6 - ≤ 6/18 in the other eye), monocular severe VI (PVA < 6/60 - ≤ 3/60 in one eye and 6/6–6/60 in the other eye) and monocular blindness (PVA < 3/60 to NLP in one eye and PVA of 6/6–3/60 in the other eye) [1].

#### Smoking

Smokers were those who smoked one stick of cigarette at least once per day and nonsmokers those who never smoke cigarette [15].

#### Eye trauma

Self-reported previous history of any trauma to the eye.

#### Family history of eye problem

Were those participants who had positive history of vision problems in their family members/near relatives (parents & grandparents).

### Data collections and examination procedures

The questionnaire was pre-tested for 5% of the sample at Chacha town which is 10 km away from Debre Berhan and re-adjusted accordingly. The questionnaire was contained socio-demographic, socio-economic, behavioral factors and ocular examinations which was used to collect the data. Ocular examinations were done by using Snellen’s “E” optotype chart, pinhole disc, pen torch, direct ophthalmoscope and 2.5 × magnifying loupe. Optometrists and Ophthalmologist were involved in the data collection process. After took the informed written consent from the study participant, Optometrists had measured the presenting distance VA at 6 m for each eye separately. Adults with VA of less than 6/18 in the worst eye were rechecked with pinhole. Presenting Visual acuity of < 6/18 in the worst eye were considered as VI. An improvement of VA with pinhole and clear ocular media with direct ophthalmoscopy was confirmed as VI due to refractive error. Both anterior and posterior segment eye examination were done for all cases to determine the possible abnormalities that decrease the VA and all the findings were documented. Those visual impaired participants who had undetermined eye problems were consulted to the Ophthalmologist for detailed eye examination and the required data were collected after the diagnosis was confirmed. All study participants who had VI were linked to the referral hospital for appropriate management and follow up.

### Statistical analysis

All the collected data was entered, coded and cleaned to EPI INFO 7 and then exported in to SPSS (Statistical Package for Social Science) version 20 and analyzed. Descriptive results were presented by using frequency, percentages, charts, tables, graphs and summary statistics. Binary logistic regression model was used to find out the association between VI and independent variables. Multivariable binary logistic regression model was used to determine the factors adjusted for potential confounders. Adjusted Odds Ratio (AOR) and 95% confidence interval (CI) were used to show the strength of association. Model fitness was checked by Hosmer and Lemeshow goodness of fit test. Multi co-linearity was checked by variable inflation factor (VIF) and tolerance. Finally, those factors with *p*-value of less than 0.05 were considered as statistically significant.

## Results

### Socio-demographic characteristics of study participants

A total of 416 participants were included in the study with a 98.6% response rate. The median age of participants was 36 years with interquartile range (IQR: 27–52 years). Among the study participants 247 (59.4%) were females (See Table 1).

**Table 1:** Socio-demographic characteristics of adults aged ≥ 18 years at Debre Berhan town, North Shewa, Ethiopia, 2018 (*n* = 416, *n =* number of study participants)

Variable	Frequency	Percentage (%)
Age (years)		
18–39	235	56.5
40–64	123	29.6
> 64	58	13.9
Gender		
Male	169	40.6
Female	247	59.4
Religion		
Orthodox	345	82.9
Muslim	27	6.5
Protestant	32	7.7
Catholic	12	2.9
Ethnicity		
Amhara	359	86.3
Oromo	27	6.5
Tigrie	13	3.1
Guragie	17	4.1
Marital status		
Single	178	42.8
Married	238	57.2
Family history of eye problem		
Yes	16	3.8
No	400	96.2

### Socio-economic characteristics of study participants

The median family monthly income was 101.07 US$ with inter quartile range of [IQR: 57.27–151.67]. Most of the study participants 354 (85.1%) had no health insurance (See Table 2).

**Table 2:** Socio-economic characteristics of adults aged ≥ 18 years at Debre Berhan town, North Shewa, Ethiopia, 2018 (*n* = 416, *n =* number of study participants)

Variables	Categories	Frequency	Percentage (%)
Health insurance	Yes	62	14.9
	No	354	85.1
Occupations	Employed	155	37.3
	Not employed	261	62.7
Educational status	Illiterate	55	13.2
	Literate	361	86.8
Family monthly income (US$)	< 57.31	106	25.5
	57.31–101.07	127	30.5
	101.11–151.61	84	20.2
	> 151.61	99	23.8

### Systemic co-morbidities and behavioral characteristics of study participants

Among the study participants 405 (97.8%) were non-smokers. History of systemic hypertension and diabetic mellitus were found in 21 (5.0%) and 9 (2.2%) participants respectively. Two hundred eighty three 68.0% of participants had no history of eye checkup (See Table 3).

**Table 3:** Systemic co-morbidity and behavioral characteristics of adults aged ≥ 18 years at Debre Berhan town, North Shewa, Ethiopia, 2018 (*n =* 416, *n =* number of study participants)

Variables	Category	Frequency	Percentage (%)
Known history of hypertension	Yes	21	5.0
	No	395	95.0
Known history of diabetes	Yes	9	2.2
	No	407	97.8
Cigarette smoking	Yes	11	2.6
	No	405	97.4
History of eye trauma	Yes	28	6.7
	No	388	93.3
History of eye check up	Yes	133	32.0
	No	283	68.0
Eye glass wear	Yes	101	24.3
	No	315	75.7

### Prevalence of visual impairment among adults

The prevalence of VI among adults aged ≥ 18 years was 16.8% [95% CI: 13.5, 20.2%]. Nearly a third 22 (31.4%) of the participants with VI were in the bilateral moderate VI category. Among the overall prevalence of visually impaired adults 27 (6.49%) had bilateral VI and 43 (10.34%) had monocular VI (See Table 4).

**Table 4:** Frequencies of VI categories among adults aged ≥ 18 years with visual impairment at Debre Berhan town, North Shewa, Ethiopia, 2018 (*n* = 70, *n =* number of adults with visual impairment)

Visual impairment category		Frequency	Percentage (%)
< 6/18–6/60	Bilateral moderate VI	22	31.4
< 6/60–3/60	Bilateral sever VI	2	2.9
< 3/60-NLP	Bilateral blindness	3	4.3
< 6/18–6/60, other eye 6/6–6/18	Monocular moderate VI	21	30.0
< 6/60–3/60, other eye 6/6–6/60	Monocular sever VI	4	5.7
< 3/60-NLP, other eye 6/6–3/60	Monocular blindness	18	25.7
Total		70	100.0

**Fig. 1:**
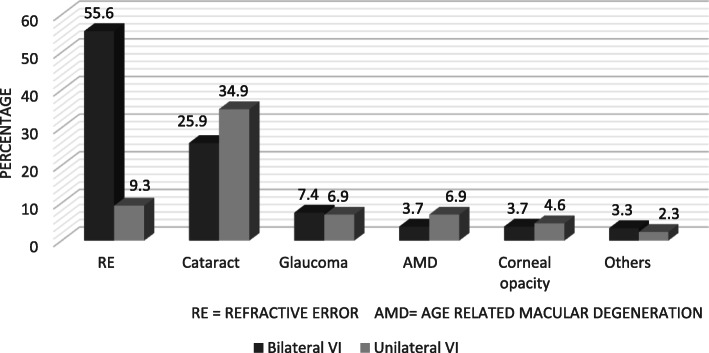
Common ocular abnormalities that caused bilateral and unilateral visual impairment among visually impaired adults aged ≥ years at Debre Berhan town, North Shewa, Ethiopia, 2018. Refractive error and cataract were the leading cause of bilateral and unilateral VI respectively

Refractive error was the most common cause of bilateral VI and cataract caused most of the unilateral VI (See Fig. 1).

### Factors associated with visual impairment in adults

In multivariable analysis age, history of eye trauma, family history of eye problem, family size and educational status had statistically significant positive association with VI.

Adults aged 40–60 years were 3 times more likely to present with VI compared to those aged 18–39 years; adults aged > 64 years were even 12 times more likely. Those who had family history of eye problems were 7 times more likely to have VI than adults with no history of family eye problems. Adults who had a history of eye trauma were 4 times more likely to have VI than those who had no previous eye trauma history. Illiterate adults were 3 times more likely to have VI than literate adults (See Table 5).

**Table 5:** Factors associated with visual impairment among adults aged ≥ 18 years at Debre Berhan town, Ethiopia, 2018 (*n* = 416, *n* = number of study participants)

Factors	Visual Impairment		COR(95% CI)	AOR(95% CI)	*p*-value
	No	Yes			
Age (year)					**0.0001**
18–39	222	13	1.0	1.0	
40–64	100	23	3.93(1.91–2.8.07)	3.04(1.22–7.58)	0.017
> 64	24	34	24.19(11.25–52.01)	12.18(4.47–33.20)	0.0001
Marital status					**0.058**
Single	141	37	1.63(0.97–2.73)	2.06(0.98–4.37)	
Married	205	33	1.0	1.0	
Education level					**0.007**
Illiterate	26	29	8.71(4.68–16.20)	3.02(1.36–6.72)	
Literate	320	41	1.0	1.0	
Family income					**0.491**
(US$)	74	32	3.85(1.78–8.35)	2.20(0.78–6.20)	0.137
< 57.31	108	19	1.57(0.69–3.54)	1.40(0.52–3.76)	0.506
57.31–101.07	75	9	1.07(0.41–2.77)	1.67(0.54–5.17)	0.373
101.11–51.61	89	10	1.0	1.0	
> 151.61					
Hypertension					**0.840**
Yes	15	6	2.07(0.77–5.53)	1.14(0.33–3.87)	
No	331	64	1.0	1.0	
Diabetes					**0.278**
Yes	5	4	4.13(1.08–15.80)	2.47(0.48–12.63)	
No	341	66	1.0	1.0	
Family history of eye problem					**0.003**
Yes	9	7	4.16(1.50–11.58)	7.02(1.95–25.22)	
No	337	63	1.0	1.0	
History of trauma					**0.003**
Yes	18	10	3.61(1.61–8.09)	4.44(1.64–12.04)	
No	328	60	1.0	1.0	
Occupation					**0.595**
Employed	141	14	1.0	1.0	
Unemployed	205	56	2.75(1.48–5.13)	1.24(0.56–2.72)	
Cigarette smoke					**0.287**
Yes	7	4	2.93(0.83–10.31)	2.42(0.42–12.29)	
No	339	66	1.0		
Family size					**0.077**
< 2	104	12	1.0	1.0	
2–4	159	30	1.64(0.80–3.34)	2.25(0.83–6.14)	**0.113**
4–5	39	9	2.00(0.78–5.12)	2.25(0.63–8.03)	**0.210**
> 5	44	19	3.74(1.68–8.36)	4.44(1.43–13.75)	**0.010**

## DISCUSION

Prevalence of visual impairment among adults aged ≥ 18 years in this study was 16.8% (95% CI: 13.5, 20.2%) which is higher than other studies done in South Sudan (11.8%) [10], Cape Town South Africa (7.2%) [16], Sokoto state of Nigeria (11%) [17], Atakunmosa, South Western Nigeria (7.4%) [18], Bangladesh (9.3%) [19], Malaysia (9.2%) [20], South Korea (4.3%) [21], East Delhi district of India (11.4%) [6], Mahabubanagar district of India (8.4%) [22], Iran (1.39%) [23] and Botucato, Brazil (7.4%) [24].

The studies in South Sudan, Sokoto and Atakunmosa, Nigerian state, Brazil, Bangladesh, Mahabubanagar district of Indian and Malaysian were done by better eye presenting visual acuity which means they considered bilateral VI only. If one eye was visually impaired and the other was not impaired, they considered as no VI which under estimate the magnitude of VI compared to the present study which considered the visual acuity of either eye. The lower prevalence of VI in Cape Town South Africa might be caused by differences in socioeconomic variables and access of eye care services. The Iran and Korean studies were based on best corrected better eye visual acuity which might under estimate the burden of VI.

The prevalence of visual impairment in this study is lower than the studies reported by Upper Egypt (38.8%) [9], Cocoa farmers of Ghana (22.7%) [8], Saudi (23.5%) [5] and rural areas of Coastal Karantaka, India (25.7%) [7].

The possible discrepancy between the studies in Upper Egypt and cocoa farmers of Ghana compared to this study might be due to study area and population difference in which they studied on rural and aged ≥40 years populations. The study in Saudi had used 6/9 as a lowest cut of point of visual acuity to define VI [5] unlike in the current study that used the lowest cut of point of visual acuity for VI was 6/18 [1] which may be the possible reason for the discrepancies.

The prevalence of visual impairment in this study is in line with the studies done in China (17.17%) [25], Andhra Pradesh state of India (14.3%) [4] and Southern Mexico (14.1%) [26]. This may be due to studying the same ages of the populations (≥18 years), use of presenting visual acuity and similar cut of point (VA < 6/18) for defining VI.

In this study, illiteracy is positively associates with visual impairment which was similar that found by other studies done in China [25], rural area of Karntaka India [27], Cape Town, South Africa [16], Southern Mexico [26]. The possible reason for this trend of VI may be poor health related behaviors in illiterates [28].

Age ≥ 40–64 years are positively associated with VI which was supported by studies done in Singapore [29], South Africa [16], China [25], South Korea [21] Western Cameroon [30], Southern Mexico [26], and Nigeria [18] that may be related to an increased prevalence of age related eye diseases and degenerations in these age groups [31].

History of trauma to the eye had 4 times more likely to have VI which can be explained due to deterioration of the eye structure, functional loss and exposure to ocular infections following trauma.

Visual impairment in adults with family history of eye problems is nearly 7 times higher than no family history which may be due to inheritance of genetic factors.

In adults within > 5 family size is nearly 4 times more likely to have VI compared to those adults within < 2 family size which can be explained due to difficulty to cater for the provision of food, health service use, education and low standard of leaving for the siblings in such large families [32].

Since community based study on the presenting visual acuity was not conducted in the study area or other similar areas which had related population characteristics and methodology of the current study, 50% proportion of sample size determination might overestimated the prevalence. In addition this study might have an inheritance limitations of the cross-sectional study design and information bias due to the tools that used to collect ocular trauma history and family ocular history. Because of the study used broad cigarette smoking categories from references different from the study area, the result might have a limitation to signify the variable in the study area.

## Conclusion

Prevalence of visual impairment was significant public health problem among adults at Debre Berhan town. Advanced age, history of eye trauma, illiteracy, large family size and family history of eye problems were positively associates with visual impairment.

## Data Availability

All the data supporting the findings are contained within the manuscript. If any additional information is required, all the necessary data will be available with the principal investigator (Natnael Lakachew Assefa with email: natiuog@gmail.com)

## Abbreviations

AOR: Adjusted Odds Ratio
CI: Confidence Interval
COR: Crude Odds Ratio
EPI INFO: Epidemiological Information
NLP: No Light Perception
PVA: Presenting Visual Acuity
SPSS: Statistical Package for Social Science
VA: Visual Acuity
VI: Visual Impairment
WHO: World Health Organization
